# Trends and characteristics of the metabolically healthy obese phenotype in an Arab population

**DOI:** 10.3389/fpubh.2024.1371359

**Published:** 2024-07-31

**Authors:** Kaiser Wani, Balvir Kumar, Nasser M. Al-Daghri, Shaun Sabico

**Affiliations:** ^1^Biochemistry Department, College of Science, King Saud University, Riyadh, Saudi Arabia; ^2^Department of Biotechnology, University Institute of Biotechnology, Chandigarh University, Mohali, India

**Keywords:** obesity, metabolically healthy obesity, chronic diseases, epidemiology, Arab population

## Abstract

The metabolically healthy obesity (MHO) phenotype represents a complex and distinctive trait, the trends and characteristics of which remain unknown in the Saudi Arabian adult population. The present study aims to fill that gap. A combined total of 10,220 Saudi adults from 2 independent cohorts [2008–2019, *N* = 7,896 (2,903 males and 4,993 females), and 2021–2023, *N* = 2,324 (830 males and 1,494 females)] aged 19–70 years old was screened, of whom 9,631 (3,428 males and 6,203 females) were included. Anthropometric data were measured, and fasting blood samples were collected to assess glucose, lipids, adipocytokines and inflammatory markers using routine methods and commercially available assays. Obesity was defined as a body mass index (BMI) ≥30 kg/m^2^. Screening for MHO was done using the empiric definition proposed by Zembic and colleagues and the by the National Cholesterol Education Program’s Adult Treatment Panel III (NCEP ATPIII). Of the 3,949 (41.0%) participants with obesity, 33.4% (95% confidence interval, CI, 32–35) were considered MHO using the empiric definition, and 32.8% (95% CI, 31–34) using NCEP-ATPIII. The overall age and gender adjusted prevalence of MHO in the Saudi adult population was 31.6% (95% CI, 30–33) and 30.1% (29–31) by the two definitions, respectively. Females had a higher age-adjusted prevalence of MHO than males (OR = 1.22, 95% CI 1.1–1.4, *p* = 0.009) as per the ATPIII criteria. MHO prevalence substantially increased over time from 2008 to 2023 (*p* < 0.001) for both definitions. Circulating leptin levels and insulin resistance were significantly higher in the MUO group than the MHO group independent of the definition used, suggesting the presence of a more severe form of leptin resistance in the MUO group which may explain the worse cardiometabolic profile as compared to the MHO group. In summary, the study highlights the first time the characteristics and trends of the MHO phenotype among Saudi Arabian adults. The pluripotent effects of leptin and its resistance may be central to MHO’s progression, or lack thereof, to the MUO phenotype, and this needs further investigation.

## 1 Introduction

Obesity is a complex public health concern that is defined by the excessive accumulation of body fat, resulting in many adverse health outcomes (1, 2). Obesity is closely associated with an elevated susceptibility to chronic health ailments such as type 2 diabetes, cardiovascular illnesses, hypertension, certain forms of cancer, and musculoskeletal problems (3–5). Furthermore, obesity-related medical expenditures are on the rise, imposing a significant economic strain on healthcare systems (6). The incidence of obesity on a worldwide scale has reached significant levels, making it a global public health issue and Saudi Arabia (SA) is not an exception (7, 8). Geographical and cultural differences need to be taken into consideration to better understand the obstacles associated with obesity in a particular region (9, 10). Therefore, public health officials, healthcare providers, and policymakers in SA need to adopt a customized approach in addressing the escalating problem of obesity (11). This approach should prioritize prevention, education, and intervention strategies that are specifically tailored to the local population (12–14).

While it is well-accepted that keeping a healthy weight may help reduce the occurrence of the adverse health consequences associated with obesity, it is becoming clear that not everyone with excess adiposity experiences the same health concerns (15, 16). The effective management of obesity requires a thorough comprehension of its multifaceted nature, including the identification of specific subgroups in obesity to devise tailored approaches for prevention and intervention (17). Significant disparities in death rates among obese persons are reported in communities where metabolic disorders, such as hypertension, diabetes, dyslipidemia, insulin resistance, or inflammatory variables, are not present (18, 19). As a result, certain adult individuals with obesity have been classified as the “metabolically healthy obese” (MHO), as they do not exhibit cardiometabolic abnormalities inherent to obesity (20–22). These individuals appear to be “protected” from the metabolic dysfunctions that are typically associated with obesity (23). MHO studies in rodent models suggest differences in the distribution of adipose tissue in response to weight gain may be responsible for the different effects on metabolic dysfunction (24). Proponents of the MHO concept argue that studying this protective mechanism could serve as a valuable foundation for developing interventions to address obesity and its related complications (25).

In contrast, alternative observations support the view that no form of obesity should be categorically labeled “healthy,” especially in the context of older adults (26). Although MHO individuals are characterized by a lack of traditional metabolic syndrome markers despite having obesity, recent research indicates that this phenotype may not be protective against long-term health risks, and MHO individuals are still at an elevated risk for obesity-related complications over time, challenging the notion that any form of obesity can be considered benign (26). The temporal aspect of metabolic health can lead to a transition from MHO to a metabolically unhealthy state (MUO). Whether MHO phenotypes are completely free of metabolic abnormalities or whether hidden metabolic complications exist that later manifest in the form of MUO phenotype, this idea of the state of MHO is an interesting subject to study. Furthermore, similar to a more variable definition and sub-grouping of diabetes (27), obesity also seems to be more heterogeneous than previously thought, and biomarkers which may be able to distinguish MUO and MHO phenotypes such as adipocytokines and inflammatory markers, would help in identifying risks for cardiometabolic complications.

To date, several definitions of MHO have been proposed (28–31) and based on these definitions, obese individuals who do not fall under the MHO category were considered as MUO. Also, the clinical characteristics and trends of MHO and MUO in the Arabian Gulf region have never been investigated, more so in SA where obesity is common among adults. This study aims to fill this gap.

## 2 Materials and methods

### 2.1 Study design and population

**Figure 1:**
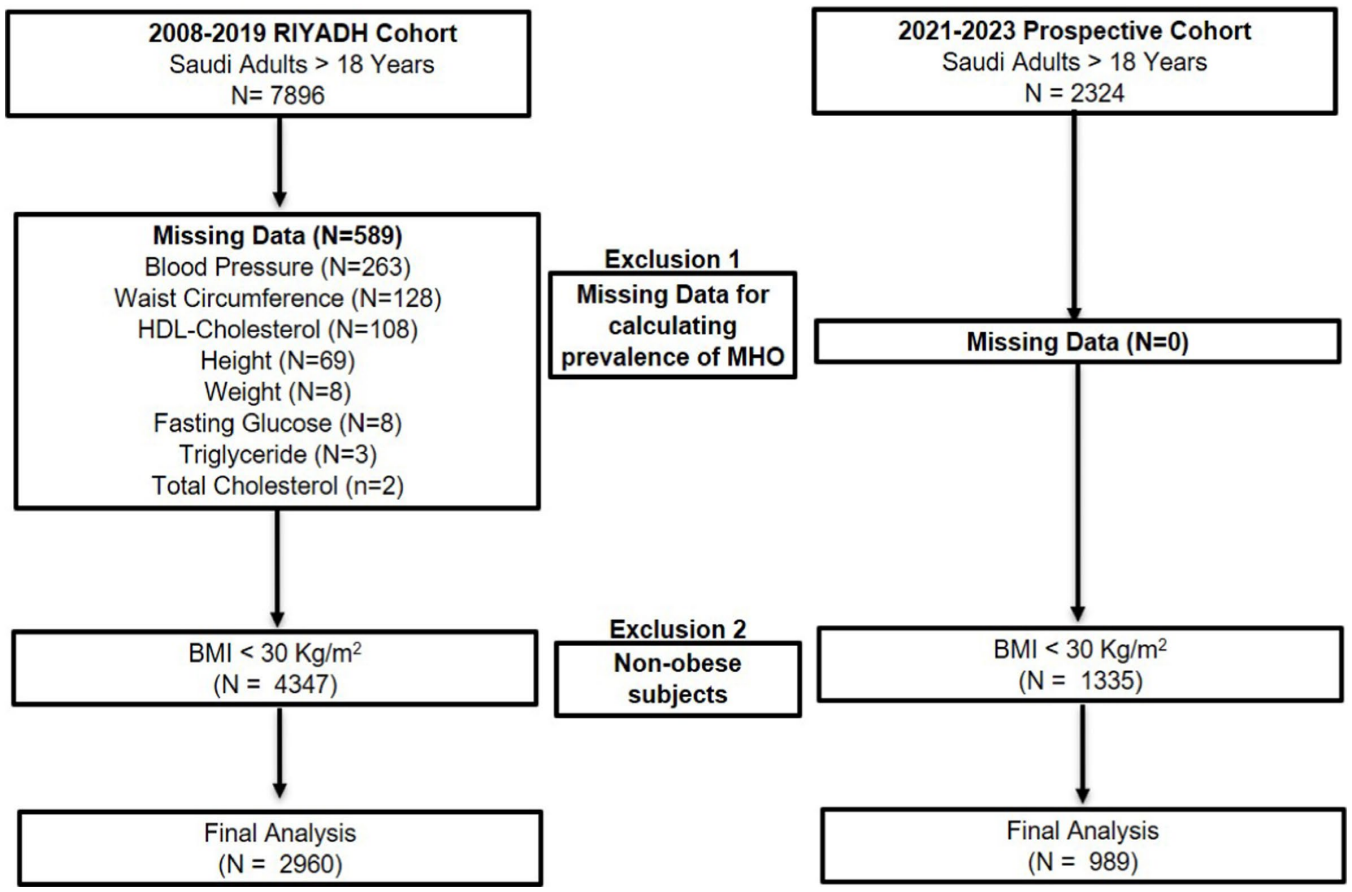
Flow chart of the sample recruitment.

In the present cross-sectional series study, two independent cohorts were used. The first cohort [*N* = 7,896, 2,903 males and 4,993 females] was taken from the master database of the Chair for Biomarkers of Chronic Diseases (CBCD) in King Saud University (KSU), Riyadh, SA collected from 2008 to 2019 (32, 33). In brief, the 2008–2019 cohort contained demographic and clinical information of almost 10,000 Saudi residents aged 7–80 years in the city of Riyadh, recruited primarily through primary care centers in collaboration with the Ministry of Health, Riyadh, SA, for use in various epidemiologic investigations (32, 33). The recruitment for the second cohort commenced in mid-2021 and extended until July 2023. During this period, a total of 2,324 consenting Saudi adults 18 and above years of age (64.3% females) were enrolled on various healthcare centers and several government-run schools in the Riyadh region. Pregnant women, those with acute and chronic conditions that required immediate medical attention, and non-ambulatory and non-consenting participants were excluded from both cohorts. The study was approved by the Institutional Review Board (IRB) at the College of Medicine, KSU (E-22-7142). Ethics approval for the Riyadh Cohort was taken from the College of Science in KSU with permission from the Ministry of Health. Figure 1 presents the detailed flow chart of the participants recruited in the study.

### 2.2 Clinical and biochemical evaluations

The database for the 2008–2019 cohort was taken from our previously published study done to investigate the prevalence of vitamin D deficiency in SA as explained in section 2.1 (33). All participants in the 2021–2023 cohort underwent a comprehensive examination, including clinical assessment, anthropometric measurements, and the collection of fasting blood samples. A standard questionnaire was administered to each participant, which included demographic information, family medical history, and individual medical history. For anthropometric assessments, standard procedures were followed, recording metrics such as weight (kg), height (cm), waist circumference, and hip circumference (cm). Resting blood pressure (mmHg) was measured twice on the right arm, at a 15-min interval using a digital portable blood pressure monitor (OMRON). The average of these two measurements was utilized for subsequent analyses. Additional derived metrics, such as the body mass index (BMI), calculated as kg/m^2^ and the waist-to-hip ratio (WHR), were also computed and documented.

Fasting blood samples were collected from each participant, processed, aliquoted, and then transported to the CBCD laboratory for biochemical evaluations. A routine biochemistry analyzer (Konelab 20XT, Thermo Scientific, Vantaa, Finland) was used to determine circulating levels of glucose, total cholesterol, HDL-cholesterol, and triglycerides, using commercially available bioassay kits (references# 981379, 981812, 981823, and 981301, respectively).

A sub-group of the obesity phenotypes was randomly selected from the two cohorts (*N* = 206 and *N* = 194 for 2008–2019 and 2021–2023 cohorts respectively) for measuring circulating adipocytokine levels including adiponectin, resistin, and leptin and inflammatory markers including tumor necrosis factor alpha (TNF-α) and C-reactive protein (CRP). To assess the circulating levels of insulin, leptin and TNF-α (kit Id: HBNMAG-51 K) and adiponectin and resistin (kit Id: HADK1MAG-61 K) in the samples, the Luminex multiplex platform (Luminexcorp, Texas) was used, enabling the simultaneous analysis of multiple biomarkers. The Homeostatic Model Assessment for Insulin Resistance (HOMA-IR) was calculated using fasting glucose and insulin values. Circulating CRP levels were determined using commercially available ELISA assay kits (My BioSource, San Diego, CA, United States; catalog numbers: MBS2505217) with intra- and inter-assay CVs of 3.95 and 6.07%, respectively.

### 2.3 Definitions used

All participants were assessed for their body mass index (BMI) status and classified as underweight (BMI < 18.5 kg/m^2^), normal weight (BMI = 18.5–24.9 kg/m^2^), overweight (BMI = 25–29.9 kg/m^2^), and obese (BMI ≥30.0 kg/m^2^) according to the standard provided by Ministry of Health, SA and recommended by World Health Organization (34). To estimate the prevalence of MHO, only Saudi adults with a BMI of ≥30 kg/m^2^ (obese) were included in the prevalence study. MHO was defined using 2 criteria: the empiric definition by Zembic et al. (30), and NCEP ATPIII (31). The empiric definition utilized three components: C1- WHR < 1.03 in males and < 0.95 in females; C2- the absence of diabetes; and C3: SBP <130 mmHG. The definition required the presence of obesity and all three components to be classified as MHO. The NCEP ATPIII definition employed five components: C1: waist circumference of ≥102 cm and ≥ 88 cm in males and females respectively; C2-HDL-cholesterol levels of <1.03 mmoL/L and < 1.29 mmoL/L in males and females respectively; C3- fasting glucose levels of ≥5.6 mmoL/L or diabetes diagnosis; C4-triglyceride levels of ≥1.7 mmoL/L; and C5- blood pressure of ≥130/85 mmoL/L. The definition required the presence of obesity and three out of five components to be classified as MHO. The criteria for the definitions used in defining MHO are presented in Supplementary Table S1.

### 2.4 Data analysis

The socio-demographic, anthropometric, and biochemical data collected for the 2021–2023 cohort was compiled with the data from the 2008–2019 cohort and analyzed using the Statistical Package for Social Sciences (SPSS) version 23.0. The categorical variables were presented as frequency (%) and the difference between the groups was calculated using the Chi-square test. Continuous variables were either presented as mean and standard deviation (SD), or median and 25th–75th percentile. Differences were tested using the parametric and non-parametric tests, respectively. A time-series prevalence of MHO was calculated by analyzing the prevalence in the 2008–2019 and 2021–2023 cohorts at various time points to observe trends over time within our study population. Sex-specific prevalence of MHO among obese subjects was adjusted with age and sex proportions from the national population provided by the general authority of statistics (35, 36) and reported. The 95% confidence intervals of the prevalence were calculated by sample proportion ± 1.96*standard error of proportion. The agreement between the two definitions of MHO used in this study was tested with the kappa-statistics. Microsoft Excel 2019 was used to plot the graphs.

## 3 Results

A total of 3,949 (1,048 males and 2,901 females) out of a total of 9,631 (3,428 males and 6,203 females) Saudi adults were included in the MHO prevalence analysis, which meant that the prevalence of obesity in the combined cohort was 41.0% (30.6% in males and 46.8% in females).

### 3.1 Characteristics of the study cohorts

The characteristics of the obese participants in the two study cohorts are presented in Table 1. In both cohorts, females were more than males (64.4% vs. 35.6 and 64.3 and 35.7% in the 2008–2019 and 2021–2023 cohorts), respectively. Among the age groups in the 2021–2023 cohort, 41-64-year-olds represented the most (59.0%) and subjects >64 years represented the least (7.3%). The distribution of the age groups was comparable to the 2018–2019 cohort. The median (range) of age variables in males and females in the whole study sample was 43 (61) and 44 (65) years, respectively. Self-reported family history and medical history of the subjects in the two study cohorts were also presented in Table 1. The 2008–2019 cohort had a comparatively higher proportion of self-reported medical conditions like diabetes, arthritis, hypertension, dyslipidemia, and asthma.

**Table 1:** Characteristics of the study population.

	2008–2019 Cohort (*N* = 2,960)	2021–2023 Cohort (*N* = 989)	*p*
Years collected			
2008–2010	634 (21.4)	**–**	*–*
2011–2013	728 (24.6)	**–**	
2014–2016	1,014 (34.3)	**–**	
2017–2019	584 (19.7)	**–**	
2021 onwards		989 (100)	
Sex			
Female	2,157 (72.9)	744 (75.2)	0.15
Male	803 (27.1)	245 (24.8)	
Age groups			
19–40 Y	1,064 (35.9)	333 (33.7)	0.43
41–64 Y	1,688 (57.0)	584 (59.0)	
>64 Y	208 (7.0)	72 (7.3)	
Demographic data^a^			
Education			
Uneducated	723 (33.6)	151 (44.9)	<0.001
Pre-college	729 (33.9)	38 (11.3)	
College	423 (19.6)	49 (14.6)	
Higher education	278 (12.9)	98 (29.2)	
Marital status			
Single	267 (12.7)	32 (9.6)	<0.001
Married	1,607 (76.4)	231 (69.2)	
Divorced	105 (5.0)	57 (17.1)	
Widowed	125 (5.9)	14 (4.2)	
Family history			
Diabetes	1,290 (69.1)	156 (72.2)	0.34
Hypertension	971 (52.9)	110 (54.7)	0.63
Dyslipidemia	144 (8.2)	20 (13.9)	0.02
Asthma	250 (14.5)	11 (8.0)	0.03
CVD	99 (5.7)	21 (14.2)	<0.001
Cancer	34 (2.0)	2 (1.5)	0.71
Medical history			
Obesity			
Diabetes	950 (34.2)	283 (28.6)	0.001
Hypertension	214 (27.1)	48 (9.7)	<0.001
Arthritis	82 (10.4)	24 (18.6)	0.007
Dyslipidemia	60 (7.6)	0 (0.0)	0.001
Asthma	114 (14.4)	0 (0.0)	<0.001
CVD	16 (2.0)	0 (0.0)	0.10
Liver disease	21 (2.7)	0 (0.0)	0.06
Kidney disease	19 (2.4)	0 (0.0)	0.08

Data was presented by N (%). The Chi-square test calculated the differences in the proportions between the two cohorts. CVD means cardiovascular disease. The superscript a represents that the demographic data was not available for all subjects and the %’s in the demographic section were calculated from those in which the data was available.

### 3.2 Clinical and biochemical characteristics according to obesity phenotypes of the study cohorts

The anthropometric and biochemical data were analyzed between the MHO and MUO obesity phenotypes and presented in Table 2. Almost all the indices in anthropometric indices like BMI, waist circumference, and systolic and diastolic blood pressure were significantly lower in MHO compared to the MUO phenotype, irrespective of the definition used (all *p*-values<0.001). The average circulating levels of fasting glucose, and triglycerides were significantly lower in the MHO group compared to the MUO (all *p*-values <0.001). Average HDL-cholesterol levels were higher in the MHO compared to the MUO group (*p* < 0.05).

**Table 2:** Clinical and biochemical characteristics in MHO vs. MUO groups.

	Empiric (30)			ATPIII (31)		
	MHO (1320)	MUO (2629)	*p*	MHO (1295)	MUO (2654)	*p*
Clinical parameters						
Age (years)	39.8 ± 11.5	48.8 ± 11.8	<0.001	40.2 ± 11.8	48.5 ± 11.9	<0.001
Weight (kg)	86.8 ± 12.8	88.5 ± 13.9	<0.001	86.0 ± 12.9	88.9 ± 13.8	<0.001
BMI (kg/m^2^)	34.2 ± 3.7	35.4 ± 4.6	<0.001	34.2 ± 3.8	35.4 ± 4.6	<0.001
WC (cm)	97.0 ± 12.3	107.3 ± 13.6	<0.001	98.5 ± 14.6	106.5 ± 13	<0.001
HC (cm)	115.0 ± 11.2	111.4 ± 14	<0.001	111.1 ± 13.2	113.3 ± 13.2	<0.001
SBP (mmHG)	114.3 ± 8.5	130.4 ± 15.9	<0.001	117.0 ± 12	129.0 ± 16	<0.001
DBP (mmHG)	73.8 ± 7.5	80.2 ± 10.3	<0.001	74.5 ± 8.3	79.8 ± 10.2	<0.001
Biochemical parameters						
T. Chol (mmol/l)	5.1 ± 1.2	5.2 ± 1.3	0.19	5.0 ± 1.1	5.2 ± 1.3	<0.001
HDL-C (mmol/l)	1.0 (0.8,1.3)	1.0 (0.8,1.2)	0.04	1.2(0.9,1.4)	0.93(0.7,1.1)	<0.001
TG (mmol/l)	1.3 (1,1.8)	1.6 (1.2,2.3)	<0.001	1.2 (0.9,1.5)	1.8 (1.3,2.4)	<0.001
FBG (mmol/l)	5.2 ± 0.9	8.2 ± 4.1	<0.001	5.0 ± 0.8	8.3 ± 4	<0.001

The data was presented as mean ± standard deviation, and median (quartile 1, quartile 3), for continuous normal, and continuous non-normal variables, respectively. Differences between the groups were calculated using Student’s *t*-test, and Mann–Whitney U test for continuous normal and continuous non-normal variables, respectively. BMI is “Body mass index,” WC is “Waist circumference,” HC is “Hip circumference,” SBP and DBP are systolic and diastolic blood pressures, T. Chol, HDL-C, LDL-C, and TG are “total cholesterol, high-density cholesterol, low-density cholesterol, and triglycerides” respectively, FBG is “fasting blood glucose.”*p* < 0.05 was considered statistically significant.

**Figure 2:**
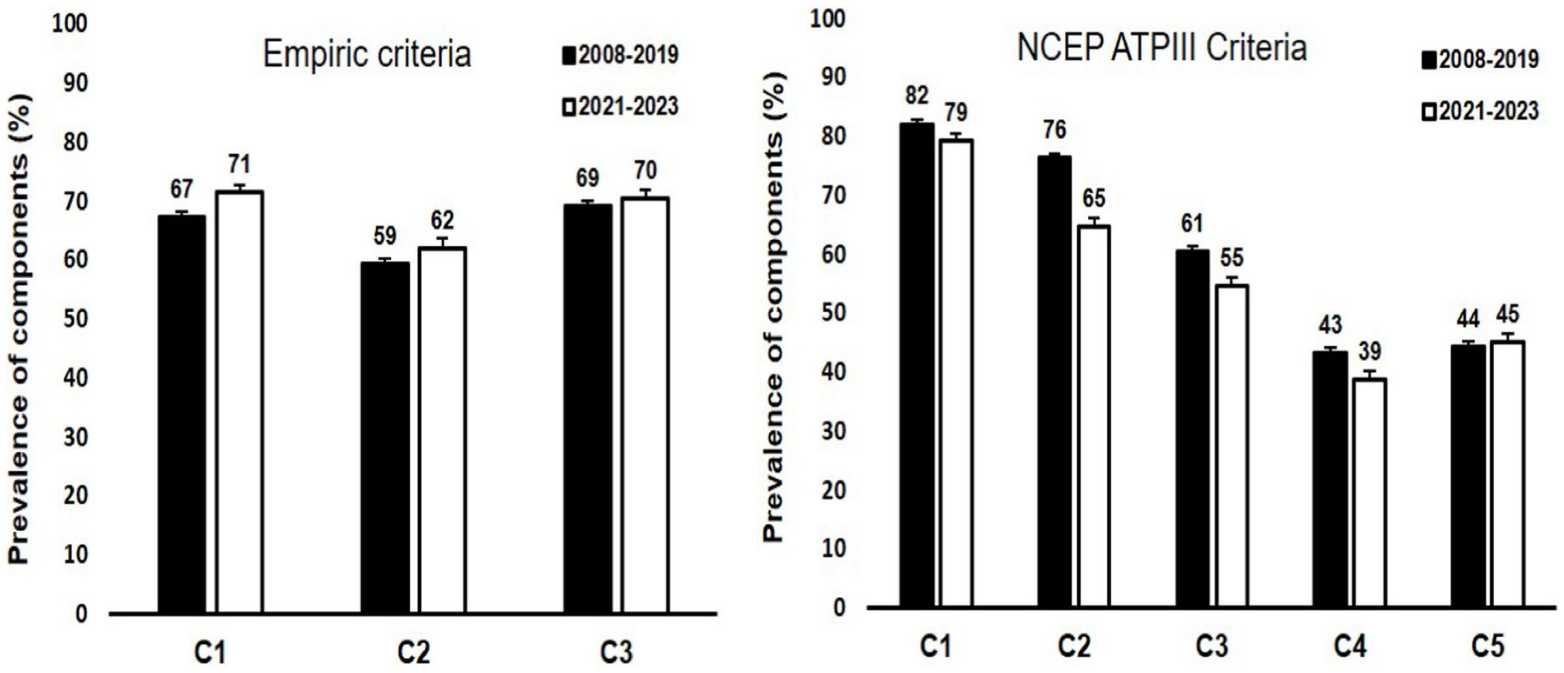
Bar graphs representing the prevalence of different components used for the two definitions of MHO in the study cohorts. The prevalence was calculated among those with obesity. For Empiric criteria, components are represented by C1: waist height ratio < 1.03 in males and < 0.95 in females; C2: absence of diabetes; and C3: systolic blood pressure < 130 mmHG, respectively. For NCEP ATPIII criteria, components are represented by C1: waist circumference of ≥102 cm and ≥ 88 cm in males and females respectively; C2: HDL-cholesterol levels of <1.03 mmoL/L and < 1.29 mmoL/L in males and females respectively; C3: fasting glucose levels of ≥5.6 mmoL/L or diabetes diagnosis; C4: triglyceride levels of ≥1.7 mmoL/L; and C5: blood pressure of ≥130/85 mmoL/L. An explanation of the two criteria has been provided in section 2.3.

The prevalence of different components used to categorize MHO according to the two definitions used has been plotted as bar graphs in Figure 2.

### 3.3 Adipocytokines and inflammatory markers according to obesity phenotypes

Both insulin and HOMA-IR were significantly higher in the MUO group than the MHO group independent of definition used. Among the adipocytokines and inflammatory markers measured, only leptin and TNF-α showed significant differences and were noted to be higher in the MUO group as compared to the MUO group, again in both definitions. No differences were seen in levels of resistin, adiponectin and CRP using the empiric definition. Interestingly, CRP levels in the MUO group was higher than the MHO group, though this significance was borderline (Table 3).

**Table 3:** Differences in adipocytokines and inflammatory markers in MHO vs. MUO groups.

	Empiric (30)			ATPIII (31)		
	MHO (*N* = 197)	MUO (*N* = 203)	*p*	MHO (*N* = 190)	MUO (*N* = 210)	*p*
Insulin (μU/mL)	8.9 (4.1,13.4)	15.8 (9.1,24.6)	<0.001	9.9 (4.1,15.2)	15.5 (9.1,26.3)	<0.001
HOMA-IR	2.3 (0.9,3.5)	4.9 (2.3,6.6)	<0.001	2.4 (1,3.6)	5.0 (2.3,7.3)	<0.001
Adiponectin (μg/mL)	17.1 (10.5,31.5)	16.6 (8.7,32.2)	0.8	18.4 (11.4,35.2)	16.3 (10.6,32.2)	0.41
Resistin (ng/mL)	23.7 (9.1,48.6)	24.4 (12.8,42.6)	0.57	23.6 (12.4,45.1)	25.0 (12.2,54.0)	0.66
Leptin (μg/mL)	3.5 (0.2,19.0)	10.4 (0.4,29.8)	0.003	3.2 (0.2,21.5)	10.0 (4.1,34.1)	0.005
TNF-α (pg/mL)	11.4 (3.5,31.9)	12.3 (5.2,27.2)	0.048	9.0 (3.0,30.3)	13.2 (5.5,28.4)	0.01
CRP (mg/dL)	0.38 (0.2,1.3)	0.59 (0.1,1.5)	0.47	0.45 (0.2,1.3)	0.48 (0.1,1.5)	0.05

The data was presented as median (quartile 1, quartile 3). Differences between the groups were calculated using the Mann–Whitney U test. HOMA is “Homeostatic Model Assessment for Insulin Resistance,” TNF is “Tumor Necrosis Factor,” and CRP is “C-reactive protein.”*p* < 0.05 was considered statistically significant.

### 3.4 Age and sex-specific prevalence of MHO

The age- and sex-specific prevalence in the two study cohorts was calculated and presented in Table 4. Out of the total 3,949 individuals with obesity in both cohorts, 1,320 (33.4%) and 1,295 (32.8%) were considered MHO by the empiric and NCEP ATPIII definitions, respectively. Kappa-statistics for the agreement between the two definitions revealed a moderate agreement (kappa statistic of 0.52). Regardless of the definition, the prevalence of MHO decreased with age in the two study cohorts with the 19-29-year group having the highest prevalence. In the 2008–2019 cohort, the prevalence of MHO among females with obesity was 33.9, and 33.6%; and in males it was 28.0 and 25.7% as per the empiric definition and by ATPIII, respectively. In the 2021–2023 cohort, similar prevalence were observed in females (33.5 and 36.4% respectively); however, in males’ higher prevalence was observed by each definition as compared to the 2008–2019 cohort (46.5 and 38.4% respectively). The prevalence of females younger than 50 and older is provided in Supplementary Table S2.

**Table 4:** Sex- and age-group-specific MHO prevalence according to different operational definitions in the BMI-defined obese population.

Age Group (years)	2008–2019 Cohort (*N* = 2,960)			2021–2023 Cohort (*N* = 989)		
	N	Empiric	ATPIII	N	Empiric	ATPIII
		(30)	(31)		(30)	(31)
Females						
19–29 years	229	148 (64.6)	152 (66.4)	49	27 (55.1)	28 (57.1)
30–39 years	427	213 (49.9)	210 (49.2)	112	68 (60.7)	69 (61.6)
40–49 years	656	212 (32.3)	201 (30.6)	183	68 (37.2)	77 (42.1)
50–59 years	591	128 (21.7)	123 (20.8)	245	61 (24.9)	76 (31.0)
60–69 years	199	26 (13.1)	33 (16.6)	126	24 (19.0)	18 (14.3)
≥70 years	55	5 (9.1)	5 (9.1)	29	1 (3.4)	3 (10.3)
*p*-value		<0.001	<0.001		<0.001	<0.001
Total	2,157	732 (33.9)	724 (33.6)	744	249 (33.5)	271 (36.4)
Males						
19–29 years	107	58 (54.2)	53 (49.5)	46	31 (67.4)	26 (56.5)
30–39 years	154	72 (46.8)	61 (39.6)	87	54 (62.1)	45 (51.7)
40–49 years	220	53 (24.1)	51 (23.2)	58	19 (32.8)	17 (29.3)
50–59 years	169	33 (19.5)	28 (16.6)	39	7 (17.9)	4 (10.3)
60–69 years	115	7 (6.1)	11 (9.6)	13	3 (23.1)	2 (15.4)
≥ 70 years	38	2 (5.3)	2 (5.3)	2	0 (0.0)	0 (0.0)
*p*-value		<0.001	<0.001		<0.001	<0.001
Total	803	225 (28.0)	206 (25.7)	245	114 (46.5)	94 (38.4)
*p* (f vs. m)		0.002	<0.001		<0.001	0.58

Data was presented as N (%). The Chi-square test calculated the differences in the proportions between the age groups and sex in the two cohorts.

**Figure 3:**
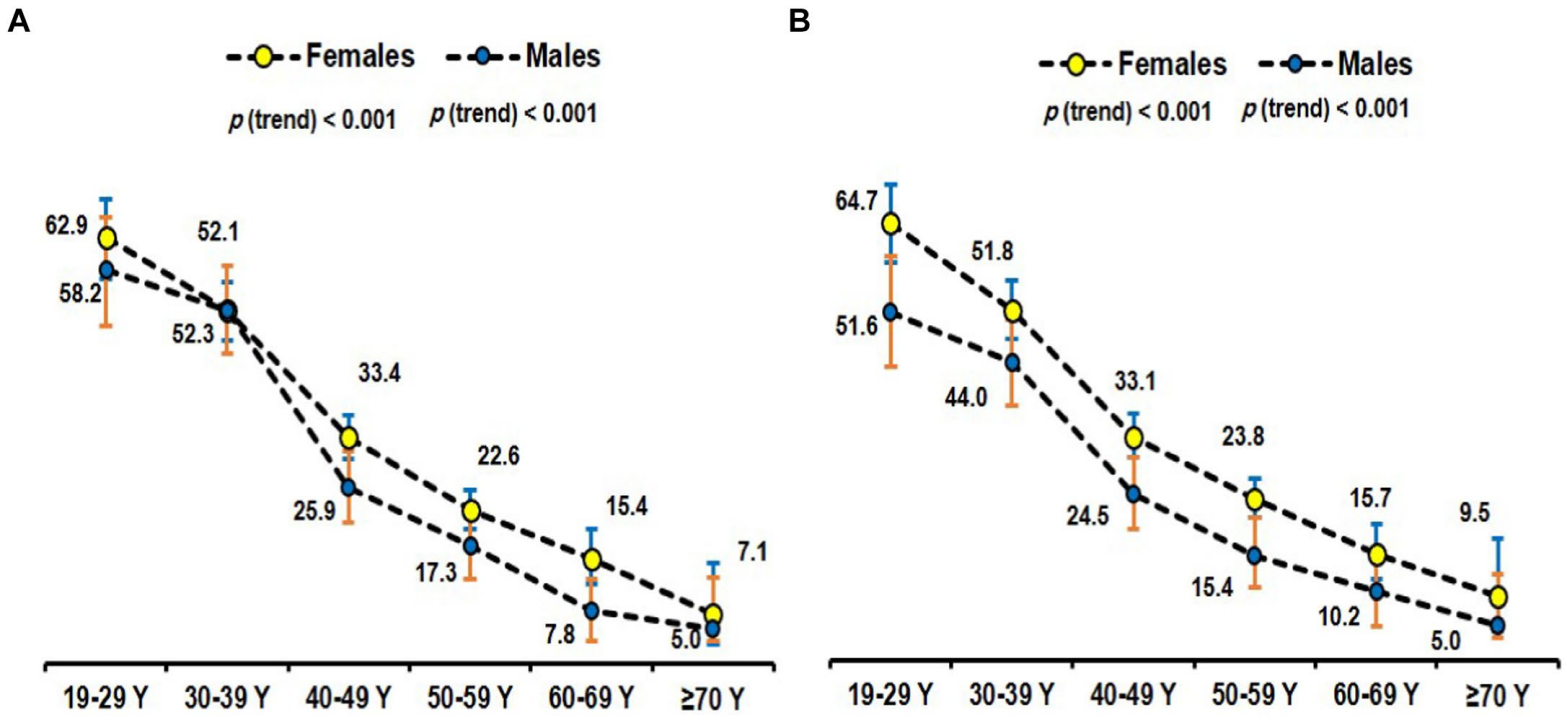
Age-specific prevalence of MHO using the empiric definition (30) **(A)** and NCEP ATP III (31) **(B)** among obese subjects.

**Figure 4:**
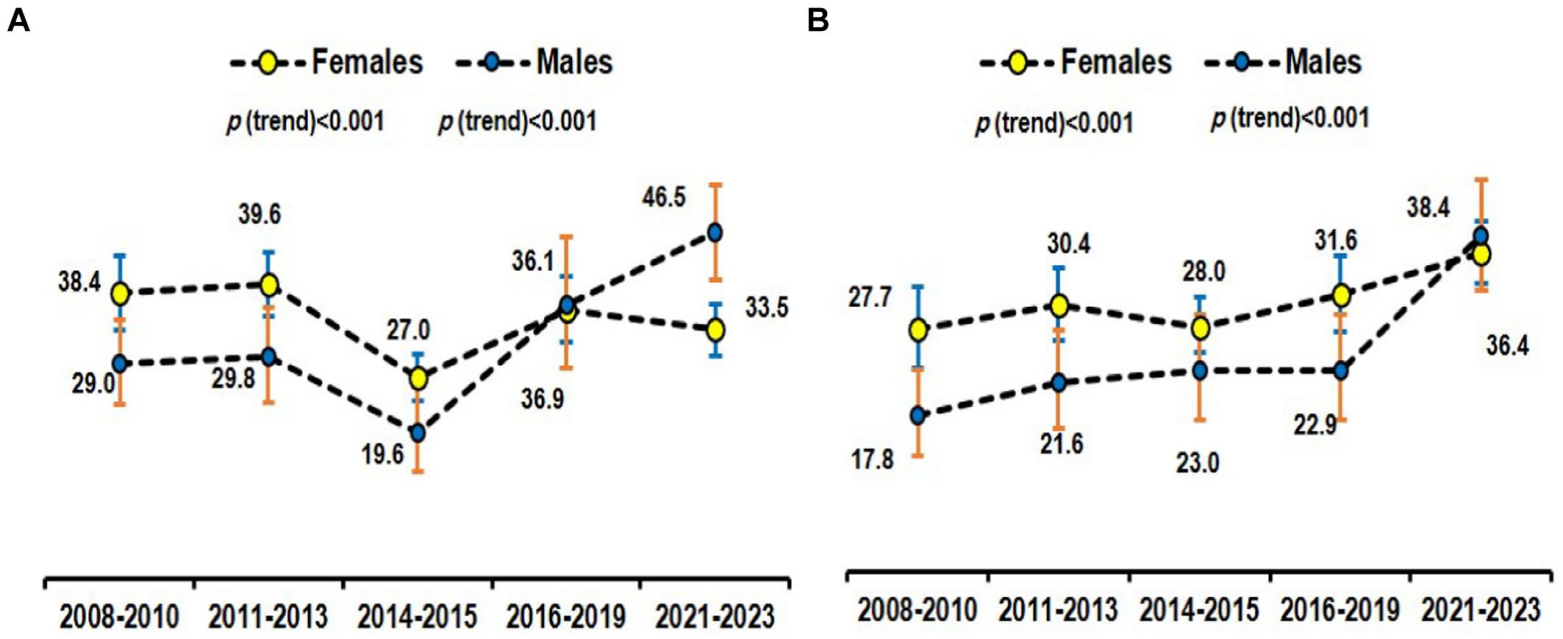
Time-series prevalence of MHO using the empiric definition (29) **(A)** and NCEP ATP III (30) **(B)** among obese subjects.

The age-specific prevalence of MHO overall according to different definitions among obese Saudi adults was plotted in Figure 3. Figure 4 provides a time-series overall prevalence of MHO according to the sample collection years which suggests an increasing trend from 2018 to 2023 in both definitions.

### 3.5 Age-adjusted prevalence of MHO

The prevalence of MHO in the study samples was adjusted with the age and sex-specific proportions from the national population statistics mentioned in the data analysis section and the resulting age and sex-adjusted prevalence was reported in Table 5. Also, the 2 × 2 chi-square contingency table for MHO among obese individuals was used to calculate the odds ratio and its 95% confidence intervals to compare the prevalence of MHO in females vs. males. The age-adjusted prevalence of MHO as per the empiric definition in females when compared to males was 36.2% vs. 37.9%, *p* = 658. The age-adjusted prevalence of MHO as per the definition of NCEP ATPIII in females when compared to males was 36.8% vs. 32.3%, *p* = 0.009. As per the NCEP ATP III definition, higher age-adjusted odds of having MHO were found in females compared to males (OR = 1.22, 95% confidence intervals of 1.1–1.4). The overall age and sex-adjusted prevalence as per empiric and NCEP ATPIII definitions was 31.6 and 30.1%, respectively.

**Table 5:** Adjusted prevalence of MHO in the study samples by different definitions.

Models		Empiric (30)	OR (95%CI), *p*	ATPIII (31)	OR (95%CI), *p*
Females vs. Males	a	33.8 (32.1–35.6)	1.07 (0.9–1.2), 0.388	34.3 (32.6–36.1)	1.30 (1.1–1.5), <0.001
		32.3 (29.5–35.3)		28.6 (25.9–31.5)	
	b	36.2 (34.5–37.9)	0.98 (0.8–1.1), 0.658	36.8 (35.1–38.6)	1.22 (1.1–1.4), 0.009
		36.9 (33.9–39.8)		32.3 (29.5–35.1)	
All subjects	a	33.4 (32.0–34.9)		32.8 (31.3–34.3)	
	c	31.6 (30.1–33.1)		30.1 (28.6–31.4)	

Data was presented as prevalence (95% CI of prevalence). Models, a, b, and c represent crude, age-adjusted, and age and sex-adjusted prevalence. The national Saudi population data was used as a reference to adjust the prevalence in the study samples. An odds ratio of having MHO among obese female vs male participants was calculated and presented as OR (95%CI). A *p* < 0.05 was considered as significant.

## 4 Discussion

The present study assessed for the first time the prevalence of the MHO phenotype in Saudi adults using two separate MHO definitions. The findings revealed that almost 40% of Saudi adults were obese, and 33.4 and 32.8% of this obese population was considered metabolically healthy by empiric and NCEP ATPIII definitions, respectively. Moreover, the age-adjusted prevalence of MHO was observed to be more prevalent among women when defined by NCEP ATPIII. The data suggests that the prevalence of MHO has an increasing overall trend from 2008 to 2023. From the cardiometabolic parameters assessed, insulin resistance and circulating leptin levels were significantly higher in the MUO group than the MHO group, suggesting that the presence of leptin resistance maybe central to the worse metabolic profile observed in the MUO phenotype. The study is arguably the first and the largest of its kind in the Arabian Gulf region to assess the trends and characteristics of MHO.

The MHO phenotype has attracted a lot of interest in the field of obesity research (37). Although obesity is often associated with several unfavorable health consequences, some individuals with obesity have a healthy metabolic profile (38, 39). Understanding MHO depends on several critical aspects, including age, sex, lifestyle, and heredity (40). Over the last 15 years, the proportion of the MHO phenotype has been reported in contradictory ways in previous publications. Several epidemiological investigations in the United States have reported MHO prevalence ranging from 29.5 to 50% among obese (41–43). In Europe, the prevalence of MHO has varied from 27.2 to 44.2% (28, 44). Studies in China and Korea have shown MHO prevalence to be 42.4 and 55.2%, respectively, when defined by NCEP ATPIII (45, 46). This variation might be partially explained by the lack of internationally accepted standards for characterizing the MHO phenotype (47).

We utilized the empiric definition (30), and NCEP ATPIII (31) criteria in our research for specific objectives. The ATPIII criterion has been the most commonly used in research of obesity phenotypes with the majority of the referenced studies having utilized it, allowing direct comparison with other reports. A newly accepted empiric definition for MHO by Zembic et al. (30) was also used in this study as this definition of MHO was not associated with total and CVD mortality in two large cohorts. Another definition of MHO, given by Wildman (28) utilizes the correlation between obesity, metabolic disorder and insulin resistance (IR) as a criterion for defining metabolic abnormality. A HOMA-IR level of ≥90th percentile in non-diabetic subjects was used as a risk factor by the criteria in Wildman. This definition was utilized extensively in the majority of research on IR and diabetes (48, 49). In addition, one of the metabolic components of the Wildman criterion was the highly sensitive C-reactive protein (hsCRP), which serves as an inflammatory marker. Nevertheless, in one study involving the Chinese population (45), elevated hsCRP levels for MHO and MUO did not differ significantly, suggesting that hsCRP may not be an essential factor in determining metabolic status.

One of the highlights of the present study was the subgroup analysis showing differences in inflammatory and adipocytokine markers, based on age- and sex-matched groups, which provided critical insights into the metabolic disparities between MUO and MHO phenotypes. We observed that the HOMA-IR levels were significantly higher in the MUO group than the MHO group across definitions. This finding aligns with previous studies suggesting that insulin resistance is a key differentiator between these obesity phenotypes (50, 51). Interestingly, while no significant differences were found in circulating adiponectin and resistin levels, our results indicated significantly higher levels of leptin and TNF-α in the MUO group compared to the MHO group under both definitions. The significantly higher leptin in the MUO group suggests the presence of leptin resistance in the MUO phenotype which was either absent or milder than the MHO group (52, 53). Leptin resistance is known not only to reduce satiety but also acts as an acute phase reactant that triggers secretion of multiple inflammatory cytokines, creating a feedback loop which promotes chronic inflammation and insulin resistance (54, 55). The presence of leptin resistance in the MUO phenotype may explain the more dysregulated metabolic and inflammatory state in this phenotype independent of obesity. It is worthy to note that leptin’s tendency for resistance and the multiple mechanisms involved made it a less ideal target in developing effective leptin analogs for treating obesity (56).

The variation of MHO prevalence in our study and also when reviewed in the literature for other populations may predominantly be attributed to the heterogeneity in the definitions of being metabolically healthy (28, 29, 31, 57). The criteria given by Zembic and colleagues utilized waist-hip ratio as a measure of central obesity along with systolic blood pressure and hyperglycemia but ignored dyslipidemia as a risk factor for metabolic disorders. One more criterion for defining MHO was given by Biobank Standardization and Harmonization for Research Excellence in the European Union (BioSHare-EU) (29) which categorizes metabolic health by the absence of the five components of metabolic syndrome which also explains the low prevalence of MHO by this criteria.

It is crucial to have a uniform criterion for defining obesity phenotypes. On the other hand, using different definitions of metabolic disorders to evaluate the forms of obesity that are more common in a given community might reduce study design variance and increase internal validity. The metric most commonly employed for assessing obesity within a population is BMI. Given this, we decided to use the BMI obesity cut-offs that the Ministry of Health, SA, had suggested (58). Furthermore, WC has been included as one of the essential elements of metabolic syndrome by the American Heart Association/National Heart, Lung, and Blood Institute and the International Diabetes Federation (59). It is however important to emphasize that while reports on the prevalence of this phenotype of obesity have utilized a wide range of criteria, only a few have looked at the impact of different criteria on the prevalence. An example is a study by Phillips et al. (60) in an Irish community using criteria such as Wildman (28), ATPIII (31), and Aguilar-Salinas (61) closely resembles the patterns we found throughout our investigation in Saudi adults.

The sexual dimorphism in MHO prevalence observed here are in line with findings from prior studies involving various populations, which have consistently demonstrated that females have a greater prevalence of MHO than men (62). Sex disparities constitute an additional noteworthy characteristic of MHO, with the variations potentially stemming from differences in the distribution of body fat and estrogen levels (63–65). Adipogenesis of subcutaneous adipose tissue is more prevalent in the gluteofemoral region of women’s lower extremities, where its influence on metabolic health is comparatively lesser, whereas visceral adipose tissue accumulation in the abdominal region is more prevalent in men (66). The presence of visceral fat is a significant indicator of cardiometabolic disorders, and research has demonstrated that estrogen levels inhibit inflammation, enhance insulin sensitivity, and prevent adipose accumulation (67, 68).

In our study, MHO was most prevalent among the younger age groups and decreased with age. Given the notable age-related variations observed in both obesity and metabolic disorders, it is conceivable that age may exert an influence on the variability in the prevalence of MHO. This variable complicates direct comparisons between studies, and hence an age-adjusted prevalence, as reported in this study, is more useful. In contrast to the age-related variation seen in this study, a study by Yoo et al. (69) did not report the age-related variation in the prevalence. One reason for this discrepancy may be that the previous study was predominantly individuals in their 30s to 40s, representing a comparatively limited age cohort. On the other hand, our research comprised a greater range of ages. Age represents a key determinant in the manifestation of MHO. Our study indicated that younger obese individuals are more likely to be metabolically healthy than their older counterparts. A partial explanation may be that as individuals age, the likelihood of accumulating metabolic risk factors increases, thereby diminishing the chances of retaining MHO status (70). Furthermore, the transition from MHO to MUO may be accelerated with age, emphasizing the importance of early intervention and lifestyle modification (71). The observed transition with age can be ascribed to alterations in the distribution of body fat; as individuals age, there is a decrease in subcutaneous fat in the lower body and an increase in abdominal fat, specifically visceral fat (72). Aging diminishes the sex disparities in visceral adipose depots, particularly among postmenopausal women (73).

Our findings revealed a notable increase in the age-adjusted prevalence of MHO among Saudi adults over the studied period (time-series analysis). This observation prompts a closer examination of the factors contributing to this rise, with a focus on lifestyle change programs and government-led counseling initiatives. The observed increase in MHO prevalence may be partially attributed to the implementation of many lifestyle change programs conducted by our research chair during the study period (13, 74–77). These programs, often designed to promote healthier eating habits, increased physical activity, and weight management, seem to have influenced the metabolic health of obese individuals positively. By addressing modifiable risk factors, such interventions could foster a metabolically healthier profile within the obese population. In interpreting this increase in the MHO prevalence over time, it is however also important to consider the design of our study. Since our analysis was based on different individuals sampled at various time points, rather than following the same cohort over time, changes in the prevalence of MHO may reflect demographic shifts or variations in the study samples and may not reflect true historical trends. Consequently, there is a need for longitudinal studies to accurately evaluate trends in MHO prevalence in this population.

Incorporating the concept of MHO in SA’s healthcare system requires a comprehensive, multifaceted approach. This approach should not only address obesity but also recognize the potential for metabolic health within the obese population. In the Saudi adult population, various factors manifest distinctively (78). SA has witnessed a surge in obesity rates, particularly among women, with nearly 40% of the adult population classified as obese (79), as also observed in this study. This poses significant public health challenges. While limited research specifically delves into MHO in Saudi adults, it is vital to explore the interplay of age, sex, lifestyle, and genetics in this unique context to develop tailored interventions that address the growing prevalence of obesity and its associated metabolic abnormalities. By understanding the multifaceted nature of MHO and its determinants, healthcare professionals and policymakers can develop targeted strategies to mitigate the health risks posed by obesity, fostering a healthier future for the Saudi population. In this context, leveraging the concept of MHO can be a strategic approach.

The authors acknowledge some limitations. The cross-sectional nature of the study limits its applicability in deciding the best criterion for defining this phenotype of obesity especially when there are no reference studies in the population. Our sample size, while significant, may not fully represent the broader Saudi population, thereby potentially limiting the external validity of our results. Besides, the dietary and physical activity data was not available which might have explained some of the findings on the diverse MHO prevalence, especially between age-groups and sex reported in this study. Nevertheless, the study is arguably the first and largest of its kind in the Arab region to determine the prevalence of MHO, highlighting an understudied obesity phenotype in an otherwise ethnically high-risk population for obesity. The inclusion of inflammatory and adipocytokines in the investigation further strengthens the study as it provides an additional layer which can advance the field forward. The study opens doors to further prospective investigations to determine factors, genetic and/or environmental, that would predispose to a healthier obesity phenotype. This should include larger and more representative samples, as well as behavioral, hormonal, biochemical, and genetic factors, in addition to pertinent cardiovascular outcomes.

In conclusion, the overall crude prevalence of MHO among Saudi adults with obesity was 33.4 and 32.8%; and age- and sex-adjusted prevalence was 31.6 and 30.1% according to the empiric and ATPIII definitions, respectively. Females had a significantly higher age-adjusted prevalence of MHO than males (OR = 1.22, 95% CI 1.1–1.4, *p* = 0.009) as per the ATPIII criterion. The substantially higher leptin levels which was parallel to the higher insulin resistance in the MUO group independent of the definition used suggests that leptin resistance, or a severe form of hyperleptinemia, may not only explain the worse cardiometabolic profile observed in the MUO phenotype, but may also be a potential target in future therapies. Continued surveillance of obesity phenotypes and longitudinal studies examining their transitions are crucial for understanding the complex interplay between obesity, metabolic health, and demographic factors.

## Data availability statement

The original contributions presented in the study are included in the article/Supplementary material, further inquiries can be directed to the corresponding author.

## Ethics statement

The studies involving humans were approved by International Ethical Guidelines were followed for the study protocol and procedures. The study was conducted according to the guidelines of the Declaration of Helsinki and approved by the Ethics Committee of the College of Medicine (E-22-7142), King Saud University, Riyadh, Kingdom of Saudi Arabia. Participants were fully informed about the purpose and procedures of the study before reading and signing the informed consent form. The studies were conducted in accordance with the local legislation and institutional requirements. The participants provided their written informed consent to participate in this study.

## Author contributions

KW: Conceptualization, Formal analysis, Methodology, Writing – original draft. BK: Supervision, Validation, Writing – review & editing. NA-D: Funding acquisition, Project administration, Writing – review & editing. SS: Conceptualization, Supervision, Writing – review & editing.
