# Supplementary material for: Trends and characteristics of the metabolically healthy obese phenotype in an Arab population
Source: Front Public Health. 2024 Jul 31;12:1371359. doi: 10.3389/fpubh.2024.1371359 (PMC11322095; doi:10.3389/fpubh.2024.1371359)
Supplement: Supplementary file 1 [file Table_1.DOCX]

**Supplementary Table S1:** Criteria for defining MHO

|  | **Empiric [1]** | **NCEP ATPIII [2]** |
| --- | --- | --- |
| BMI (≥30 Kg/m^2^) | ✓ | |
| Criteria for MHO | plus | |
|  | All of below | ≤2 of 5 components below |
| Waist Circumference (cm) | | |
| Male ≥102 |  | ✓ |
| Female ≥88 |  |  |
| Waist Hip Ratio (WHR) | | |
| Male <1.03 | ✓ |  |
| Female<0.95 |  |  |
| Fasting Blood Glucose (mmol/l) | | |
| ≥ 5.6 or Diabetes Medications or Diagnosis |  | ✓ |
| No Diabetes | ✓ |  |
| Blood Pressure (mmHG) | | |
| ≥ 130/85 |  | ✓ |
| Systolic BP<130 mmHG | ✓ |  |
| Blood Pressure Medications |  |  |
| Diagnosis for CVD |  |  |
| Fasting Triglycerides (mmol/l) | | |
| ≥1.7 |  | ✓ |
| High Density Lipoprotein (mmol/l) | | |
| Male <1.03 |  | ✓ |
| Female<1.29 |  |  |

**Supplementary Table S2:** Prevalence of MHO among females with obesity

| **Age Group (years)** |  | | |  | | |
| --- | --- | --- | --- | --- | --- | --- |
|  | **2008-2019 Cohort (N=2960)** | | | **Prospective Cohort (N=989)** | | |
|  | **N** | **Empiric** | **ATPIII** | **N** | **Empiric** | **ATPIII** |
|  |  |  |  |  |  |  |
| **FEMALES** | | | | | | |
| <51 years | 1473 | 607 (41.2) | 597 (40.5) | 358 | 171 (44.3) | 184 (47.7) |
| >50 years | 684 | 125 (18.3) | 127 (18.6) | 386 | 78 (21.8) | 87 (24.3) |
| *p-value* | | <0.001 | <0.001 |  | <0.001 | <0.001 |
| Total | 2157 | 732 (33.9) | 724 (33.6) | 744 | 249 (33.5) | 271 (36.4) |

**^Note:^** The Data was presented by N (%). The Chi-square test calculated the differences in the proportions between the age groups and gender in the two cohorts.

1. Zembic A, Eckel N, Stefan N, Baudry J, Schulze MB. An empirically derived definition of metabolically healthy obesity based on risk of cardiovascular and total mortality. JAMA Network Open. 2021; 4(5):e218505-e218505.

2. National Cholesterol Education Program . Expert Panel on Detection, Treatment of High Blood Cholesterol in Adults,Third report of the National Cholesterol Education Program (NCEP) Expert Panel on detection, evaluation, and treatment of high blood cholesterol in adults (Adult Treatment Panel III). 2002(2).
